# Antifungal Activity and Phytochemical Screening of Vernonia amygdalina Extract against Botrytis cinerea Causing Gray Mold Disease on Tomato Fruits

**DOI:** 10.3390/biology9090286

**Published:** 2020-09-11

**Authors:** Siti Fairuz Yusoff, Farah Farhanah Haron, Mahmud Tengku Muda Mohamed, Norhayu Asib, Siti Zaharah Sakimin, Faizah Abu Kassim, Siti Izera Ismail

**Affiliations:** 1 Department of Crop Science, Faculty of Agriculture, Universiti Putra Malaysia, Serdang 43400, Selangor, Malaysia; yuezyusoff@gmail.com (S.F.Y.); mtm59@gmail.com (M.T.M.M.); szaharah@upm.edu.my (S.Z.S.); 2 Agricultural Science Department, Faculty of Technical and Vocational, Universiti Pendidikan Sultan Idris, Tanjong Malim 35900, Perak, Malaysia; faizah@ftv.upsi.edu.my; 3 Pest and Disease Management Programme, Horticulture Research Centre, Malaysian Agricultural Research and Development Institute, Serdang 43400, Selangor, Malaysia; farahfarhanah@mardi.gov.my; 4 Department of Plant Protection, Faculty of Agriculture, Universiti Putra Malaysia, Serdang 43400, Selangor, Malaysia; norhayuasib@upm.edu.my

**Keywords:** *Vernonia amygdalina*, antifungal activity, *Botrytis cinerea*, phytochemical, tomato, gray mold disease

## Abstract

Gray mold disease caused by *Botrytis cinerea* is a damaging postharvest disease in tomato plants, and it is known to be a limiting factor in tomato production. This study aimed to evaluate antifungal activities of *Vernonia amygdalina* leaf extracts against *B. cinerea* and to screen the phytochemical compound in the crude extract that had the highest antifungal activity. In this study, crude extracts of hexane, dichloromethane, methanol, and water extracts with concentration levels at 100, 200, 300, 400, and 500 mg/mL were shown to significantly affect the inhibition of *B. cinerea.* Among the crude extracts, dichloromethane extract was shown to be the most potent in terms of antifungal activities. The SEM observation proved that the treatment altered the fungal morphology, which leads to fungal growth inhibition. For the in vivo bioassay, the fruits treated with dichloromethane extract at 400 and 500 mg/mL showed the lowest disease incidence with mild severity of infection. There were 23 chemical compounds identified in *V. amygdalina* dichloromethane extract using GCMS analysis. The top five major compounds were dominated by squalene (16.92%), phytol (15.05%), triacontane (11.31%), heptacosane (7.14%), and neophytadiene (6.28%). Some of these significant compounds possess high antifungal activities. This study proved that *V. amygdalina* from dichloromethane extract could be useful for inhibiting gray mold disease on tomato fruit and has potential as a natural antifungal agent.

## 1. Introduction

In Malaysia, approximately 96.30% of tomato production comes from the highlands, including Lojing, Kelantan, and Cameron Highland, Pahang [1]. The temperatures of these two locations range from 18 to 22 °C, with a relative humidity of 93–95%, which are optimal conditions for the development of fungal pathogen. *Botrytis cinerea* is a fungal pathogen of gray mold disease that can infect dicotyledonous plants, including tomato. Subjected to scientific and economic importance, *B. cinerea* was ranked as the second top plant pathogen in the world [2]. In 2015, Tijjani et al. [3] observed tomato fruits with gray mold symptoms in postharvest storage at Cameron Highlands with 65% disease incidence, and this is the first report of gray mold disease on tomato fruits in Malaysia.

*B. cinerea* infection can develop in the field and can also cause postharvest decay or remain latent until storage. Spore germination of this pathogen grows vigorously in higher relative humidity and low temperature [4]. Thus, in cold storage, it leads to the development of gray mold symptoms, and this disease spreads rapidly among fruits in the same packaging. Chemical fungicides are the most commonly used to control gray mold on tomato fruits, but the repeated use of synthetic fungicide can develop fungal resistance and be harmful to consumer health. A few modes of action from a new fungicide provide adequate protection for fresh tomatoes [5]. However, the residue and toxicity concerns may limit their use.

Among the postharvest strategies in controlling plant diseases, natural products offer a promising treatment to reduce the disease incidence of postharvest diseases. Natural products contain advanced chemical novelty compared to chemically synthesized products, and for this reason, researchers try to discover new bioactive compounds in plants [6]. Plant extracts also contain beneficial secondary metabolites such as phenolics, tannins, coumarins, quinones, flavonoids, saponins, terpenoids, and alkaloids. These compounds have been proven to be potentially significant in plant protection as antimicrobial agents [7]. Many experiments have been conducted using plant extracts to control *B. cinerea* pathogen, which causes gray mold disease. Soylu et al. [8] found that essential oils extracted from rosemary and lavender could cause hyphae shriveled, protoplast leakage, conidia loss, and cytoplasmic coagulated on *B. cinerea* morphology. The extracted essential oil of fennel, cinnamon, and anis have also shown fungicidal effects on *B. cinerea* in in vitro and in vivo tests [9]. Moreover, the extraction of oregano and lemon effectively lowered the disease severity of gray mold disease in tomatoes, strawberries, and cucumbers [10]. In recent findings, stilbene extracted from grapevine leaves possessed antifungal activity of *B. cinerea* by inhibiting the mycelium growth and simultaneously reducing the necrotic lesion [11].

Bitter leaf is scientifically known as *Vernonia amygdalina*. In Africa and Asia, it is commonly used as a medicinal plant [12]. Various parts of *V. amygdalina,* including the leaf, root, and stem have been used for their antidiabetic, antioxidant, antimicrobial, anticancer, anti-inflammatory, and antiplasmodial effects [13]. Among the plant parts, researchers identified that the leaf part accumulates the highest chemical constituents and nutritional compositions [14]. Detailed investigations in the compound purification of *V. amygdalina* extract discovered many promising active compounds; for example, flavonoids, triterpenoids, saponins, tannins, sesquiterpene lactones, alkaloids, terpenes, phenolics, and steroidal glycosides [12]. According to Akowuah et al. [15], the extract from *V. amygdalina* was non-toxic in mice when exposed to up to 2000 mg/kg/day for 28 days.

To date, most researchers have focused on *V. amygdalina* crude extract in order to uncover its potential as an antifungal agent for the management of plant disease. Recent findings found that *V. amygdalina* ethanol extracts showed a good ability to inhibit postharvest fungal pathogens *Rhizopus stolonifer* and *Fusarium moniliforme* [16,17]. In another study, in an in vitro test using an ethanol crude extract of *V. amygdalina* at 300 mg/mL, the growth of *Cercosporella persica* and *Curvularia lunatus* were completely inhibited [18]. However, there has been no report on the antifungal activity of *V. amygdalina* extracted from different polarities of solvent against *B. cinerea* isolated from tomato. Therefore, this study aimed to (a) evaluate in vitro antifungal activities of *V. amygdalina* crude extract against *B. cinerea* in tomato; (b) study the effect of *V. amygdalina* extract on the morphology of *B. cinerea*; (c) determine in vivo antifungal activities of *V. amygdalina* crude extract against *B. cinerea*; and (d) screen the chemical constituents in *V. amygdalina* extract that are responsible for antifungal activities.

## 2. Materials and Methods

### 2.1. Plant Materials

Mature plants of *V. amygdalina* were collected from the local supplier in Puchong, Selangor and verified by a botanist from the Biodiversity Unit, Institute of Bioscience, Universiti Putra Malaysia. A voucher specimen (SK 3280/18) was deposited in the herbarium of the same institute. The plants were thoroughly cleaned, and the stems were cut into 25 cm lengths and planted in Ladang 15, Universiti Putra Malaysia (geographical coordinates: 2°59′19.8″ North, 101°43′50″ East, Malaysia) in December 2017. After two months, approximately 17 kg of matured leaves were harvested and brought to the Postharvest Laboratory, Universiti Putra Malaysia. The leaves were rinsed under running tap water to remove dirt, shade-dried for one week, and oven-dried at 40 °C for 4 h. The dried leaves were ground for two minutes using a high-speed grinder. The powdered sample was kept in an airtight container for further extraction processes.

### 2.2. Preparation of V. amygdalina Crude Extracts

**Figure 1:**
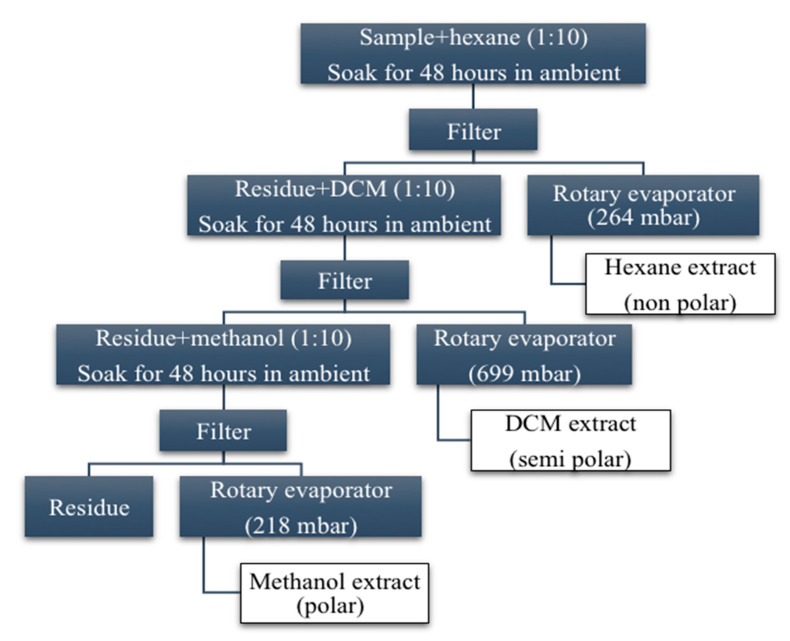
Sequential extraction procedure of *V. amygdalina.*

The organic solvents in analytical grade (99% minimum purity) of hexane (Bendosen Laboratory Chemicals, Shah Alam, Malaysia), dichloromethane (DCM, Macron-Fine Chemicals, France), methanol (HmbG Chemicals, Hamburg, Germany), and acetone (Bendosen Laboratory Chemicals, Shah Alam, Malaysia) were used in the crude extraction process. The sequential extractions were performed according to the method as described by Haron et al. [19]. The sequential extraction procedure was shown in Figure 1. Each of the extracts was concentrated in a Buchi rotary evaporator until a sticky dark green crude extract was obtained. The highest percentage of crude extract yield was methanol (15.34%), followed by dichloromethane (DCM) (4.40%) and hexane (2.62% g). The crude extracts were kept in an airtight jar and stored at 4 °C.

For the aqueous extract, 1 kg of the sample was soaked in 10 L of sterile distilled water and sonicated for 30 min at 50 °C. Then, it was filtered by using filter paper. The filtration was stored at –80 °C in a deep freezer (Ultra Low Temperature Freezer DW-86L386, Haier Medical and Laboratory Co., Ltd., Shandong, China). Next, it was freeze-dried in a freeze dryer (Christ Freeze Dryer BETA 1-8LD, Martin Christ Gefriertrocknungsanlagen GmbH, Osterode am Harz, Germany) for three days. The obtained crude extract was ground using a pestle and mortar to obtain a 389.5 g fine powder and kept at 4 °C for further use.

### 2.3. Preparation of B. cinerea

*B. cinerea* was isolated from the diseased tomato fruits. The pure culture of the fungus was sub-cultured in potato dextrose agar (PDA) media. The identification of this specific fungus was viewed under a microscope for morphology characteristics. The morphological and molecular characteristics of *B. cinerea* were similar to the description of Javed et al. [20] and Tijjani et al. [3].

### 2.4. In vitro Evaluation for V. amygdalina Antifungal Activity

The crude extract was dissolved in 1 mL of acetone and sterilized using 0.4 μm syringe filter (Sartorius). Next, the extract solution was mixed with 15 mL of PDA in a sterile vial. The mixture was vortexed thoroughly and poured into an 85-mm Petri dish. The agar in Petri dishes was allowed to solidify to become poison agar. Poison agar medium was prepared individually at the concentration of 100, 200, 300, 400, and 500 mg/mL for each crude extract. Mycelial plugs from the pure culture of *B. cinerea* with 5-mm diameters were transferred to the center of the Petri dish containing PDA. Then, the plates were put into incubator (Model LM-575RD, Yihder Technology Co. LTD, Taipei, Taiwan) and incubated at 20 ± 2 °C for eight days to produce full-plate growth of mycelium with conidia. The edge of the active growing fungal plug (4 mm) from *B. cinerea* was taken and placed at the center of poison agar. The Petri dishes were incubated up to 8 days in an incubator (20 °C), and the fungal growth was observed. The percentage of radial growth (*PIRG*) was calculated after eight days of incubation as follows.

$$PIRG=\frac{R1-R2}{R1}\;\times 100$$

*R*1 and *R*2 are radial growth of fungus for control and extract, respectively.

### 2.5. Microscopic Observation Using a Scanning Electron Microscope (SEM)

The plates that contained the highest in vitro antifungal activities were viewed under SEM to confirm the fungal inhibition. The plugs (1 × 1 cm) were harvested from the control and DCM-treated plates, respectively. The sample preparation protocol followed that described by Heckman et al. [21]. Each plug was fixed in 2.5% glutaraldehyde for five hours at 4 °C. Next, the plugs were washed with 0.1 M sodium cacodylate buffer for three changes of 10 min each. One percent of osmium tetroxide was used in the post-fixed process for two hours at 4 °C. Then, the plugs were rewashed with 0.1 M sodium cacodylate buffer for three changes of 10 min each. A series of acetone was applied every 10-min interval in the dehydration process (35%, 50%, 75%, 95%) including 100% acetone for three changes every 15 min. The specimens were transferred into a specimen basket and put into a critical dryer for 1.5 h. After that, all specimens were stuck onto the stub and sputter-coated (Baltec SCD005) with gold in an ion sputter for two minutes. All sample specimens were viewed by microscope examination using JEOL JSM-6400 SEM.

### 2.6. Antifungal Activities of V. amygdalina by In Vivo Bioassay

The fresh and premium quality of tomato fruits in maturity index 3 (green with slight red) was selected. Two hundred of the selected fruits were washed under running tap water and air-dried for two hours. Then, the fruits were sprayed with 70% (*v*/*v*) ethanol and air-dried in laminar flow at ambient temperature for 30 min. Next, 120 fruits were dipped into the three most effective extract solutions that showed the highest antifungal activity from in vitro bioassay (DCM at 300, 400, and 500 mg/mL) for five minutes. For negative control, 40 fruits were dipped in sterile distilled water and acetone. Meanwhile, another 40 fruits that were dipped in 0.5g/L Kenlate fungicide solution (active ingredient: 50% w/w benomyl) were used as a positive control. All treated fruits were excised using sterile cork borer (2 mm deep and 5 mm wide) at the equatorial side.

The preparation method of fungal plugs for in vivo bioassay was similar to that for the in vitro bioassay. The fresh fungal plug at 4 mm was harvested using a cork borer at the outermost layer of mycelium and inserted at the excised treated fruits. Finally, the fruits were kept in plastic boxes (40 fruits/box) and stored in ambient temperature at 95% relative humidity for five days [22]. Each treatment was replicated four times. Each replication consisted of ten fruits, and the experiment was repeated twice. At the end of storage duration, disease incidence and disease severity index (DSI) were determined using the following equations.

$$Disease\;incidence\;\left(\%\right)=\;\frac{Number\;of\;infected\;fruits}{Total\;number\;of\;fruit\;per\;treatment}\;\times 100$$

$$DSI=\;\overset{}{\underset{}{\sum }}\frac{a\;\times n}{AB}\;\times 100$$

where *a* = disease scale, *n* = number of fruits in a specific scale, *A* = highest disease scale, *B* = total number of fruits.

The disease severity was evaluated based on scale 0 to 4 as described by Rosero-Hernández et al. [23] using the following scale: 0 = no visible symptoms of fruits (no infection); 1 = 1–25% inoculated area covered with slight necrotic and water-soaked lesion (mild infection); 2 = 26–50% of the inoculated area covered with necrotic, white to gray mycelia and water-soaked lesion (moderate infection); 3 = 51–75% of fruits are necrotic with spore mass appeared and water-soaked (severe infection); 4 = >76% necrotic tissue appears soft, watery and decayed (very severe).

### 2.7. Screening for V. amygdalina Chemical Constituents Using Gas Chromatography-Mass Spectrometry (GCMS) Analysis

The crude samples at 0.02 g were dissolved in 1 mL acetone (HPLC grade, Fisher Chemical, USA). The solution was vortexed and filtered using a 0.02 μm filter syringe (Sartouris). The sterile crude extract was inserted into the HPLC vial and analyzed using GC-MS QP2010 Ultra (Shimadzu, Kyoto, Japan) comprising a gas chromatograph interfaced with a mass spectrometer. The compound separation was carried out using a Rxi-5MS fused silica capillary column of 30 m × 0.25 mm internal diameter (di) and 0.25 mm in film thickness (Restek GmbH, Homburg, Germany). The conditions for analysis were set as follows: column oven temperature was programmed column oven temperature at 50 °C, injection temperature maintained at 250 °C, injection mode split, flow control mode at the pressure of 37.1 kpa, total flow of 11.8 mL/min at 1 mL/min, column flow of 0.8 mL/min, split ratio of 10.0, ion source temperature 200 °C, interface temperature 250 °C, solvent cut time 2.0 min, and detector gain mode by relative and detector gain 0.88 kV + 0.00 kV. The column oven temperature was set at 50 °C (maintained for 3 min), raised at 10 °C/min to 280 °C (maintained for 3 min), and finally maintained at 300 °C for 10 min. Mass spectra were taken at the start time of 2.5 min and end time of 93.0 min. The ACQ mode Scan was carried out at an event time of 0.10 s, with a scan speed of 10,000 m/s. The start m/z was 40, and the end m/z was 700. Constituents were identified based on data libraries by analyzing and comparing mass spectra (FFNSC1.3.lib, WILEY229.lib and NIST11s.lib).

### 2.8. Experimental Design and Statistical Analysis

For the in vitro bioassay, the experiment was conducted in completely randomized design (CRD) with four replications. The test was arranged in two factorial analysis consisting of 4 types of crude extracts (hexane, DCM, methanol, aqueous) × 5 concentration levels (100, 200, 300, 400, and 500 mg/mL). For the in vivo bioassay, the experimental design was also in CRD and four replications, thus leading to a total of 80 experiments. The data were analyzed using analysis of variance (ANOVA), and the means were separated using the least significant difference (LSD) test at *p* ≤ 0.05. The data analysis was carried out in SAS software (version 9.4).

## 3. Results

### 3.1. In Vitro Antifungal Activities of V. amygdalina Crude Extract against B. cinerea

In this study, PIRG was measured and calculated to determine the antifungal activity of four types of crude extracts at five different concentrations. As shown in Table 1, both the main factors of the crude extracts and the concentration levels significantly inhibited the in vitro growth of *B. cinerea*. There was also a highly significant interaction effect between crude extract and concentration levels (CE × CL) on *B. cinerea* growth.

**Figure 2:**
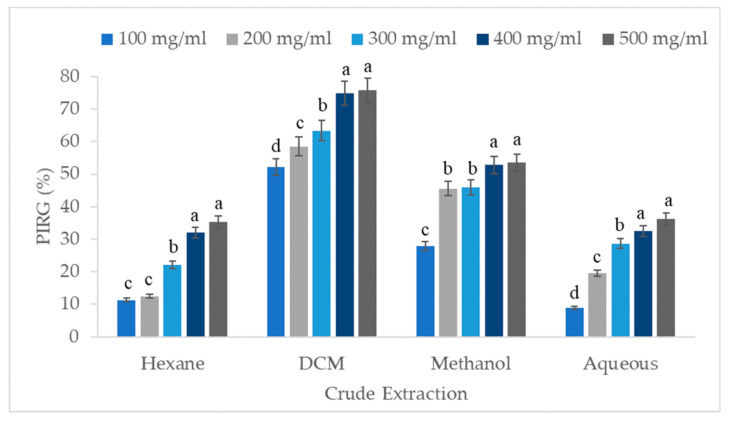
Effect of crude extraction of *V. amygdalina* at various concentrations on the PIRG of *B. cinerea* after eight days of incubation. Means with the same letter within each crude extraction are not significantly different at *p* ≤ 0.05 using the least significant difference (LSD) test.

The results indicate that the DCM crude extract of *V. amygdalina* possessed the most potent antifungal activity, exhibiting a fungistatic effect on the growth of *B. cinerea* followed by methanol, aqueous, and hexane crude extract (Figure 2). As expected, higher concentrations of 400 and 500 mg/mL showed the highest PIRG of *B. cinerea* for all crude extracts. However, both concentration levels showed no significant differences in *B. cinerea* inhibition growth. The effects of PIRG of *B. cinerea* between methanol crude extract at 200 and 300 mg/mL as well as hexane crude extract at 100 and 200 mg/mL were not significant. Within the concentration level of DCM extract, the PIRG increased as the concentration level was raised to 400 mg/mL. This result shows that the maximum inhibitory effects of *B. cinerea* radial growth were DCM at 400 and 500 mg/mL, with 74.85% and 75.7% inhibition, respectively.

### 3.2. Effect of V. amygdalina Crude Extract on the Morphology of B. cinerea

**Figure 3:**
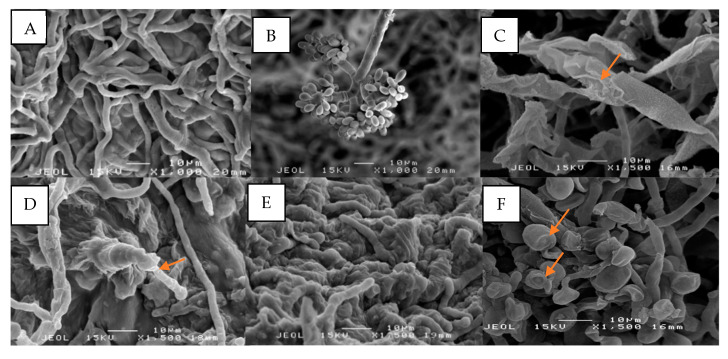
Effects of dichloromethane (DCM) crude extract on *B. cinerea* at 400 and 500 mg/mL on mycelium morphology viewed under SEM. (**A**) Healthy mycelium are slender and uniform, with a smooth surface and an intact structure in the control plate; (**B**) Healthy conidiophore from the control plate; (**C**) Mycelia were ruptured, folded with edge burrs, and sheet-like structure at 400 mg/mL; (**D**) The hyphae tip was wrinkled and deformed at 400 mg/mL; (**E**) Agglutinated mycelia at 500 mg/mL; (**F**) The conidia were shrunken at 400 mg/mL.

The morphology of *B. cinerea* was altered after being exposed to *V. amygdalina* DCM treatment. Under SEM observation, the mycelia of this fungus were shriveled, retarded, and agglutinated while the conidia underwent shrinkage (Figure 3C–F) compared to the control, which had a slender shape, and was massive, and for which the conidiophore was grape-shaped with ellipsoidal conidia (Figure 3A,B).

### 3.3. In Vivo Antifungal Activities of V. amygdalina Crude Extract against B. cinerea

The in vivo experiment showed that all fruits from the negative control were infected with gray mold disease with 100% of disease incidence (Table 2). Meanwhile, the artificially inoculated tomato fruits that were dipped in *V. amygdalina* DCM treatment at 400 and 500 mg/mL had reduced disease incidence of gray mold by 50% and 53.13% compared to the control fruits, respectively. However, the effect on the disease incidence at both concentrations was not significantly different.

Artificially inoculated tomato that was treated with *V. amygdalina* DCM extract showed significantly lower disease incidence compared to the commercial fungicide (benomyl) treatment. Regarding the DCM treatments, DCM at 400 mg/mL could reduce incidence by 2.23% compared with DCM at 300 mg/mL.

Among the infected fruits, the disease severity displayed a different pattern according to the treatment. Table 3 indicated that the fruits in negative control were observed 27.28% in disease severity index, with a value of 2 for severity scale and moderate infection category. The necrotic tissue in control fruits was covered with white to gray mycelia and water-soaked lesions.

The tomato fruits that were treated with *V. amygdalina* DCM extract at concentration 300–500 mg/mL with severity scale 1 were shown to have a significantly lower percentage of disease severity compared to chemical fungicide (benomyl). Thus, the treatment of *V. amygdalina* DCM extract could be an alternative fungicide, constituting a natural antifungal agent against the gray mold disease of tomato fruits. However, nonetheless, the gray mold disease severity of all treated fruits of DCM extract treatments and benomyl constituted a mild infection.

### 3.4. Phytochemical Screening of DCM Crude Extract

**Figure 4:**
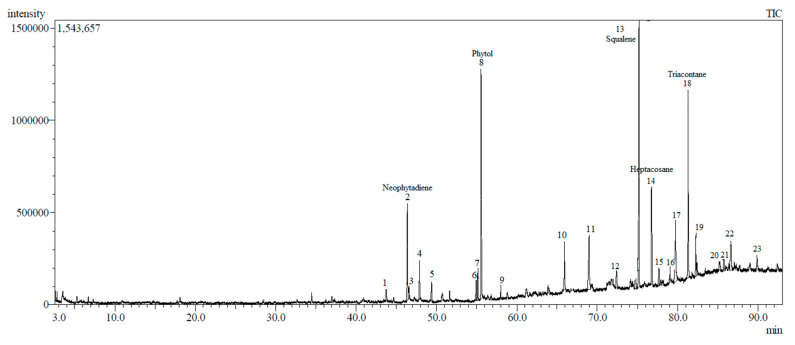
Ion chromatogram of DCM crude extract using GCMS.

GCMS analyses of the crude extract led to the identification of 23 chemical constituents in the DCM crude extract of *V. amygdalina* (Figure 4).

The identified compounds are arranged according to their elution order on silica capillary columns. The extract contains a complex mixture consisting of mainly triterpenoid, diterpene alcohol, sesquiterpene, and hydrocarbon lipid. The top five major compounds are squalene (16.92%), phytol (15.05%), triacontane (11.31%), heptacosane (7.14%), and neophytadiene (6.28%) (Table 4).

The other characteristic constituents of the crude extract are loliolide, phytone, 2-hexadecen-1-ol, 3,7,11,15-tetramethyl-2-hexadecen-1-ol, hexadecanoic acid, 9,12-octadecadienoic acid, linolenic acid, caryophyllene, γ-elemene, 1,3,7-nonatriene-1, geranyl linalool, tetratriacontane, α-tocopherol, stigmasterol, α-spinasterol acetate, chondrillasterol, α-tocopherol acetate, and one unknown compound were found to be minor components of DCM *V. amygdalina* leaves extract in the present study. Chemical residue of solvents used in the extraction was not found by GCMS analysis.

## 4. Discussion

Extraction methods involve the separation of the active compound of plant tissues from inactive components using selective solvents. During extraction, solutions defuse into solid plant material and solubilize compounds with similar polarity. The present experiments showed that extracts obtained from *V. amygdalina* contain essential components for the inhibition of mycelial growth of *B. cinerea* pathogenic on tomato plants. However, each of the crude extracts was varied in terms of their antifungal activities. Among the crude extract, DCM extract (semi-polar) of *V. amygdalina* showed the most potent inhibition on *B. cinerea* growth. As reported in other cases [24], DCM extract fraction of *Pseudognaphalium robustum* reduced the in vitro mycelial growth of *B. cinerea* at 45.5 μg/mL by 50%. They stated that it also affected the conidial germination of the *B. cinerea* by reducing oxygen consumption and interrupting plasma membrane integrity.

In contrast, Righini et al. [25] found that the water extract of *Anabaena* sp., *Ecklonia* sp., and *Jania* sp. inhibited the in vitro growth of *B. cinerea* at 2.5, 5.0, and 10.0 mg/mL. Generally, it appears that the inhibitory effect of the plant extracts varies depending on the specific plant and solvent used with no specific trend related to the polarity of the solvent. The usage of different solvent systems will extract a diversity of molecules with distinct polarities. For instance, methanol tends to extract a diversity of compound groups such as polyphenols, glycosides, and flavonoids [26], which can contribute to the fungal inhibitory effect of the extract. DCM and hexane tend to extract mainly semi-polar and nonpolar constituents such as terpenoids, fats, and fatty acid [27]. It would appear that both polar and nonpolar constituents contributed to the antifungal activity of the plant extracts.

The percentage of fungal inhibition in amended PDA medium was also dependent on concentration, and the most significant reduction in mycelial growth was obtained with the highest concentration of crude extract. A similar result was observed by other researchers using ethanol leaf extract of *V. amygdalina* against tomato diseases [16]. This could be due to the level of composition antifungal compounds obtained from the crude extract. The inhibitory effect of DCM extract increased as the concentration increased, showing more than 50% growth inhibition at all concentrations. However, the inhibition growth of *B. cinerea* showed no significant difference in any crude extract at concentrations of 400 or 500 mg/mL. A possible reason for this circumstance is the low water solubility of the antifungal compounds, which limits the miscibility in the agar medium through the poison agar method. Meanwhile, the hydrocarbon components either remained on the medium surface or evaporated, depending on its nature. According to Krzyśko-Łupicka et al. [28], the biocidal action from the plant extract also depends on the chemical composition, concentration, and phytopathogenic fungi strains.

In vivo experimental results show that negative control fruits were 100% infected with gray mold disease with a severity value of 2. The necrotic tissue in negative control fruits was water-soaked and covered with fungal mycelia. The highest *Botrytis* incidence was observed in tomatoes six days after inoculation [29], and in kiwi seven days post-inoculation [30]. Meanwhile, the artificially inoculated tomato fruits that were dipped with the two highest concentrations of DCM extract resulted in the highest reduction in disease incidence of gray mold. The possible reason for these results is the concentration level of antifungal compounds from plant extract being sufficient to control the inoculum of *B. cinerea* on tomato fruits. This statement is supported by the disease severity of gray mold on tomato in the present study, which only causes a mild infection on the fruits. The results provide baseline information for the potential use of the crude extract in the treatment of postharvest gray mold disease. This was necessary because this disease was a latent field infection [31]. However, the treatment data we obtained did not prevent the onset of gray mold disease because the percentage of disease incidence reduction was not 100%, although it was generally low. Fillinger and Elad [32] suggested that *B. cinerea* is very challenging to control due to its broad host range, different mode infection, and both asexual and sexual stages enabling it to survive in favorable or unfavorable conditions.

Meanwhile, it is well known that the fungicide activity of benomyl on a broad spectrum of phytopathogenic fungi was due to its ability to be absorbed by the phytopathogen cells. However, the present study found that benomyl treatment on *B. cinerea* has a fungistatic activity. Benomyl has been applied as a systemic fungicide since 1970 and was used to control *B. cinerea* in 1971 [33]. Methyl 2-benzimidazole carbamate (MBC) is the major metabolite of benomyl and is primarily responsible for the fungitoxicity. Hammerschlag and Sisler [34] indicated that the primary metabolites were inhibited by the synthesis of DNA, interrupted fungal cell division, and inhibited cytokinesis. However, in this case, the benomyl treatment failed to act as a fungicidal. This could be due to the fungal resistance to this fungicide, since benomyl has been used for many years.

In the present study, the highest antifungal activity of DCM extract against *B. cinerea* could be associated with the presence of 23 bioactive compounds identified using GCMS. The major compounds were dominated by squalene (16.92%), phytol (15.05%), triacontane (11.31%), heptacosane (7.14%), and neophytadiene (6.28%). Squalene is the most abundant in this crude extract. It is a naturally occurring triterpenoid and a precursor for the synthesis of secondary metabolites such as sterols, hormones, or vitamins. Squalene has been shown to have excellent antioxidant, anticancer, antibacterial, and antifungal biological activities [35]. In pharmacognosy, squalene is extensively used as an excipient for disease management and therapy [36]. Gnamusch et al. [37] found that squalene at high concentration resulted in disturbances in the fungus cellular membranes and interfered with essential membrane functions. The non-toxic chemical nature of lipids makes them excellent carriers as well as their ability to permeate the cell membrane of fungus due to their lipidic nature.

Phytol is the second-highest compound in this extract. It was classified in the diterpene group. Haque and co-workers [38] explained that terpenoids from the plant extract reduce the mitochondrial content of fungus, which could alter the ATP generation and level of reactive oxygen species. Consequently, the mitochondria of the fungus become dysfunctional. Yoshihiro et al. [39] reported that diterpene of phytol could disrupt the cell membranes of the fungus, resulting in K+ ions leaking from the cells, and causing the fungus hyphae to wither.

The aliphatic hydrocarbons of triacontane (n-C30) and heptacosane (n-C27) are identified as major components in this extract. Both long-chain alkanes were found on the surface of plant parts and in abundance in the epicuticular wax of matured leaves [40]. They acted as a physical barrier on the plant in terms of water loss, irradiation, phytopathogen attack, and insect herbivores. The appearance of the epidermal wax of the *V. amygdalina* leaf surface was proven by Eltahir and AbuEReish [41]. They reported that under SEM observation, the epidermal wax is present in large quantities at the abaxial leaf part compared to the adaxial part. Yin et al. [42] found that the hydrocarbon waxes inhibited *Alternaria* rot of pear by stunting the spore germination and mycelial growth of *Alternaria alternata*. However, to date, the specific mechanisms of action of these hydrocarbon compounds as antifungal agents against phytopathogenic fungi have been less reported.

Neophytadiene is a sesquiterpene compound and was found to be a significant component in DCM extract. Neophytadiene is an active compound with antibacterial, antifungal, antipyretic, and antioxidant activities [43]. This sesquiterpene compound could pass through the cell wall, interrupt the cell membrane function, and destroy the fungal mitochondria structure [44]. In a previous study, the sesquiterpene compound isolated from *Magnolia grandiflora* was also proven to have fungicidal effects against *A. alternata* and *F. culmorium* [45]. Neophytadiene extracted from *Daucus carota* subsp. *sativus* was identified as a major compound and showed protective as well as preventive activity against *B. cinerea* in strawberry [46].

On the other hand, some of the chemical constituents that appear in lower amounts in this extract, such as terpenoid, steroid, and fatty acid, might also contribute to the antifungal activity. Howard et al. [47] stated that *V. amygdalina* contains bioactive sesquiterpene lactones that possess highly antifungal effects. Similarly, Ivanescu et al. [48] explained that the mechanism of biological activity of alkylating sesquiterpene lactones and the nucleophile sulfhydryl group in proteins led to the disruption of cell function that caused cell wall damage of the fungus. It is possible that the minor chemical constituents might also be related to synergism effects with major compounds, inhibiting *B. cinerea* growth.

The mechanism action of antifungal compounds of the *V. amygdalina* extract on the fungal inhibition was observed under SEM. The hyphae of *B. cinerea* exposed to the phytochemical compounds of *V. amygdalina* extract revealed alterations in the hyphal morphology. The mycelia became twisted and folded with a jagged edge. Some mycelia were agglutinated, with withered hyphae tips. Another important observation was the shrinkage of conidia after the treatment. This could prevent the dispersion of the gray mold disease of fruits to the adjacent fruits, since the asexual spores of *B. cinerea* are abundant and easily dispersed by wind or water. The morphology alteration of fungus was related to the secondary metabolites from the plant extract that acted as antifungal substances to restrict the fungal growth [49]. This mode of action was called antibiosis. The antibiosis happened when the secondary metabolites from the plant extract inhibited or restricted the growth of the pathogen. The secondary metabolites inhibited fungal growth through cell membrane disruption, cell wall synthesis inhibition, mitochondrial dysfunction, cell division inhibition, protein synthesis inhibition, and efflux pump inhibition [32]. In this case, the composition of major antifungal compounds, including squalene, phytol and neophytadiene, as well as minor antifungal compounds, acted as synergistic effects in controlling the *B. cinerea* development.

## 5. Conclusions

This study provides evidence that *V. amygdalina* extracts of DCM at 400 and 500 mg/mL had the highest antifungal activities against *B. cinerea* through in vitro and in vivo bioassays. The treatment altered the fungal morphology and inhibited fungal growth. The chemical constituents in this plant extract have the potential to be a natural antifungal agent. Thus, we propose an alternative disease management strategy using *V. amygdalina* extract to control gray mold disease on tomato. However, further research is necessary to elucidate the mechanism of action and develop the formulation to improve its efficacy and stability for use in postharvest disease control. The postharvest quality study should also involve a formulation application to make sure the quality of fresh fruits is optimal and that the fruits are safe to consume.

## Figures and Tables

**Table 1:** Main and interaction effects of *V. amygdalina* crude extracts and concentration level on PIRG of *B. cinerea*. PIRG: percentage of radial growth.

Factors	PIRG (%)
Crude extracts (CE)	
Hexane	22.65 ± 2.34d ^x^
Dichloromethane	64.94 ± 2.19a
Methanol	45.15 ± 2.28b
Aqueous	25.18 ± 2.32c
Concentration levels (mg/mL) (CL)	
100	25.07 ± 4.47d
200	34.04 ± 4.93c
300	40.04 ± 4.25b
400	48.05 ± 4.61a
500	50.18 ± 4.29a
Significance	
CE × CL	**

Values are expressed as mean ± SD. ^X^ Means with the same letters within a column and each factor are not significantly different at *p* ≤ 0.05 using the LSD test. ** *p* ≤ 0.01.

**Table 2:** Percentage of *B. cinerea* incidence on tomato treated fruits.

Treatment	Disease Incidence (%)
Negative control	100 ± 0.00a ^X^
Benomyl	68.75 ± 2.69b
DCM 300 mg/mL	60.41 ± 2.69c
DCM 400 mg/mL	50.0 ± 3.40d
DCM 500 mg/mL	46.88 ± 3.13d

Values are expressed as mean ± SD. ^X^ Means with the same letters are not significantly different at *p* ≤ 0.05 using the LSD test.

**Table 3:** Percentage of disease severity index on tomato treated fruits.

Treatment	DSI (%)
Negative control	27.28 ± 0.29a ^X^
Benomyl	10.84 ± 0.69b
DCM 300 mg/mL	4.50 ± 0.53c
DCM 400 mg/mL	2.27 ± 0.12d
DCM 500 mg/mL	2.19 ± 0.05d

Values are expressed as mean ± SD. ^X^ Means with the same letters are not significantly different at *p* ≤ 0.05 using the LSD test.

**Table 4:** Chemical composition in DCM crude extract of *V. amygdalina.*

Peak	Retention Index **	Compound Name	Chemical Group	Area (%)
1	1763	Loliolide	Monoterpenoid hydroxylactones	0.76
2	1839	Neophytadiene	Sesquiterpene	6.28
3	1841	Phytone	Terpene ketone	0.90
4	1860	2-Hexadecen-1-ol	Acyclic diterpene	0.79
5	1882	3,7,11,15-tetramethyl-2-hexadecen-1-ol	Acyclic diterpene	1.46
6	1966	Hexadecanoic acid	Fatty acid	1.06
7	2089	9,12-Octadecadienoic acid	Fatty acid	1.19
8	2119	Phytol	Diterpene alcohol	15.05
9	2147	Linolenic acid	Fatty acid	1.86
10	2499	l-caryophyllene	Bicyclic sesquiterpene	3.35
11	2577	γ-Elemene	Sesquiterpene	5.72
12	2711	1,3,7-Nonatriene-1	Monoterpene	1.35
13	2830	Squalene	Triterpene	16.92
14	2892	Heptacosane	Hydrocarbon lipid	7.14
15	2577	Geranyl linalool	Monoterpenoid	1.12
16	2993	Tetratriacontane	Hydrocarbon lipid	1.02
17	3021	Unknown	-	6.41
18	3097	Triacontane	Hydrocarbon lipid	11.31
19	3139	α-Tocopherol	Vitamin E	6.04
20	3272	Stigmasterol	Stigmastane	0.98
21	3290	α-spinasterol acetate	Stigmastane	1.37
22	3295	Chondrillasterol	Triterpene (sterol)	2.90
23	3472	α-tocopherol acetate	Vitamin E	1.46

** Retention index on the Rxi-5MS silica capillary column.
